# Representative Elementary Length to Measure Soil Mass Attenuation Coefficient

**DOI:** 10.1155/2014/584871

**Published:** 2014-01-30

**Authors:** J. A. R. Borges, L. F. Pires, J. C. Costa

**Affiliations:** Laboratory of Soil Physics and Environmental Sciences, Department of Physics, State University of Ponta Grossa, Avenue Carlos Cavalcanti 4748, 84030-900 Ponta Grossa, PR, Brazil

## Abstract

With increasing demand for better yield in agricultural areas, soil physical property representative measurements are more and more essential. Nuclear techniques such as computerized tomography (CT) and gamma-ray attenuation (GAT) have been widely employed with this purpose. The soil mass attenuation coefficient (*μ*_*s*_) is an important parameter for CT and GAT analysis. When experimentally determined (*μ*_es_), the use of suitable sized samples enable to evaluate it precisely, as well as to reduce measurement time and costs. This study investigated the representative elementary length (REL) of sandy and clayey soils for *μ*_es_ measurements. Two radioactive sources were employed (^241^Am and ^137^Cs), three collimators (2–4 mm diameters), and 14 thickness (*x*) samples (2–15 cm). Results indicated ideal thickness intervals of 12–15 and 2–4 cm for the sources ^137^Cs and ^241^Am, respectively. The application of such results in representative elementary area (REA) evaluations in clayey soil clods via CT indicated that *μ*_es_ average values obtained for *x* > 4 cm and source ^241^Am might induce to the use of samples which are not large enough for soil bulk density evaluations (*ρ*_*s*_). As a consequence, *ρ*_*s*_ might be under- or overestimated, generating inaccurate conclusions about the physical quality of the soil under study.

## 1. Introduction

A representative elementary size (RES) corresponds to the volume, area, or length of a sample, necessary to provide measurements which represent the whole [1]. When studying soil, the use of samples with representative size is highly relevant due to the dependence that the physical properties of these porous media present in relation to their sizes [2]. Samples which do not present RES might generate high standard deviation values and might not be representative of the spatial structure, hampering measurements [3].

Computerized tomography (CT) and gamma-ray attenuation (GAT) techniques have been successfully applied to the determination of the soil physical properties [4, 5]. Both supply measurements in a noninvasive way, once they are based on the principle of interaction of radiation with matter.

The soil mass attenuation coefficient (*μ*_*s*_) is an important parameter to characterize the penetration into and interaction of radiation with these porous media [6]. Accurate *μ*_*s*_ measurements are important to obtain representative results of physical properties analyzed via CT and GAT. In this study, the soil mass attenuation coefficient experimentally determined was named *μ*_es_.

Despite the *μ*_*s*_ dependence being only related to the media chemical composition and the incident beam photon energy, in experimental measurements it is subject to variations associated with the measurement [7, 8]. Even good geometry suited to restrictions for the use of Beer-Lambert law might cause effects which result in under- or overestimated *μ*_es_ values.

Some studies show the *μ*_es_ dependence on factors such as sample thickness and the size of the collimator utilized. The use of samples with the ideal thickness might minimize errors due to the photons multiple scattering [9, 10]. Larger collimators increase the acceptance angle at the detector face which, in turn, result in detection of a higher number of scattered photons [11, 12]. However, few analyses such as those are available in the literature related to soil. Due to its varied composition, higher complexity is associated with these porous media, when compared to homogeneous ones.

This study aims to define the representative elementary length (REL) in *μ*_es_ measurements of two soils with different texture. The analysis was carried out employing photons with the most commonly used energy in soil science (^241^Am and ^137^Cs) and three different diameters of circular collimators (2–4 mm).

## 2. Material and Methods

### 2.1. Sample Collection and Preparation

Two experimental areas belonging to the “Escola Superior de Agricultura Luiz de Queiroz” (ESALQ), SP, Brazil, (22°40′S, 47°34′W, and 580 m above sea level) were made available for the study. Samples were collected at the superficial layer (0–10 cm) from two soils with different texture: sandy loam (770 g kg^−1^ sand, 50 g kg^−1^ silt, and 180 g kg^−1^ clay) and clay (240 g kg^−1^ sand, 330 g kg^−1^ silt, and 430 g kg^−1^ clay).

Samples were dried in oven at 105°C for two days and sieved through 1 mm sieve. This procedure was carried out in order to obtain more homogeneous filling of samples in the acrylic containers used for the *μ*_es_ measurements.

### 2.2. Elemental Analysis

The elemental analysis was carried out with a dispersive energy X-ray fluorescence (XRF) spectrometer, Shimadzu, model EDX-720. This equipment has in its tube the element rhodium as target; its voltage ranges from 5 to 50 kV and the filament operates with 1 to 1000 *μ*A currents. A Si(Li) semiconductor which works under cooling at −196°C through liquid nitrogen was used as detector. The primary filters are Zr, Ni, Ti, and Al.

Samples were ground in a mortar and then placed in the sample case, which was sealed in the lower and upper parts with 6 *μ*m thick mylar. Five replications were performed for each soil, and approximately 2 g of soil was used for each measurement.

XRF spectrum was obtained for each sample in a time of 100 s and energy bands Na-Sc (15 kV) and Ti-U (50 kV). All measurements were carried out with pressure below 30 Pa (vacuum).

These results of elemental analysis were used to calculate theoretical values of *μ*_*s*_ via the program XCOM [13, 14]. A similar procedure was carried out to determine the water mass attenuation coefficient (*μ*_*w*_).

### 2.3. Soil Mass Attenuation Coefficient Measurements

The gamma spectrometer used has a measurement table composed of a Pb castle which has radioactive sources of ^137^Cs (661.6 keV) and ^241^Am (59.54 keV). A NaI(Tl) plain type detector (7.62 cm × 7.62 cm) was used to detect the gamma photons.

Counting times adopted for the *μ*_es_ measurements were 600 s and 1200 s for the ^137^Cs and ^241^Am sources, respectively. Due to this time being considered relatively high, the room background was monitored daily with the same counting times. In order to do that, the source output was blocked with a “blind” collimator and a 10 cm thick lead block.

At the detector input, a collimator was fixed with 4.5 mm diameter for all measurements, while at the source output it was possible to fix collimators of different diameters. The laboratory temperature was kept around 19 ± 1°C.

Daily spectra of radiation were measured throughout the whole experimental procedure, which made it possible to adjust the photopeak windows during the measurements. A 2 mm diameter collimator and counting times of 30 s (^137^Cs) and 60 s (^241^Am) were employed in the spectra measurements for the free beam and with each of the two samples used. The distance between source and detector was kept fixed and equal to 23 cm. The photopeak FWHM (full width at half maximum) was monitored daily.

The *μ*_es_ was determined from the Beer-Lambert law:

(1) $$\begin{array}{c} \mu_{\text{es}}=\frac{1}{x\rho_{s}}\ln\left(\frac{I_{0}}{I}\right), \end{array}$$

where *I*_0_ and *I* (counts per second) represent the incident and transmitted intensities of photons through the samples, *ρ*_*s*_ (g cm^−3^) is the soil bulk density, and *x* (cm) is the thickness..

The error of *μ*_es_ measurements was obtained using the following relation:

(2) $$\begin{array}{c} d\mu_{\text{es}}=\frac{1}{x\rho_{s}}\left(\frac{\sqrt{I_{0}}}{I_{0}}-\frac{\sqrt{I}}{I}\right). \end{array}$$

Three circular collimators with 2, 3, and 4 mm diameter were used. *μ*_es_ measurements were carried out in different size samples. For that, 14 acrylic boxes were built with thickness varying from 2 to 15 cm. All boxes had width and height of approximately 7.0 and 6.5 cm, respectively. They were filled carefully in order to keep the samples density constant as much as possible at 1.39 ± 0.01 g cm^−3^ for the sandy soil and 1.24 ± 0.01 g cm^−3^ for the clayey one.

Throughout the measurements, each box was placed in front of the source output, touching the collimator, so that the beam could go approximately through the center of the sample and orthogonally to this position. The distance between source and detector was kept the same throughout the measurements.

### 2.4. Data Analysis

The best *ρ*_*s*_ results are usually obtained from soil samples with thickness below 10 cm with the radioactive source ^241^Am and 10–25 cm for the ^137^Cs [6]. Based on these results, in this study the *μ*_es_ average values were calculated for thicknesses *x* < 10 and *x* ≥ 10 cm. This procedure was carried out with the results of each soil, collimator, and source employed. Results were compared to the theoretical value (XCOM), through the relative difference (RD) between them.

*μ*_es_ average values were utilized as reference to verify the occurrence of measurement stabilization with different sample thickness used. The *μ*_es_ of soils with similar texture presented coefficient of variation (CV) around 2% [6]. Thus, the following criteria were adopted to establish the REL:

- relative difference between the *μ*_es_ value of each sample thickness used and its average value (*x* < 10 and *x* ≥ 10 cm) not superior to 2%;
- at least three consecutive thicknesses cannot differ from each other regarding *μ*_es_ values, employing the variation criterion in item (i).

### 2.5. REL and Soil Bulk Density Measurements

Results of *μ*_es_ measurements obtained with the ^241^Am source and clayey soil were applied to representative elementary area (REA) analyses for the *ρ*_*s*_ measurements via CT. For that, tomographic data of 18 clod samples of the clayey soil were employed.

#### 2.5.1. CT Scanner

First generation tomography equipment was used, with fixed source and detector and rotation and translation movements of the sample. The tomograph is equipped with a ^241^Am (59.54 keV) gamma-ray source with activity of about 3.7 GBq and NaI(Tl) 7.62 cm × 7.62 cm detector. The lead collimators used at the source output and detector input were 1 mm and 4.5 mm, respectively. The tomography unit (TU) matrices obtained were 80 × 80 for all tomography. The resolution obtained for the clod samples was 1.1 × 1.1 mm^2^ and the tomography system linear steps were 1.0 to 1.1 mm. A 2D image was obtained of each clod with the scanning being performed at the center of the sample. Further details about the equipment can be found in Pires et al. [4] and Cruvinel et al. [15].

#### 2.5.2. Obtaining the Soil Bulk Density Matrix

Each pixel of the tomographic image has a characteristic TU value, which is proportional to the linear attenuation coefficient of the media. For soils, the TU corresponds to the contribution of the mineral particles, organic matter, water, and air [16]. The relation between TU and *ρ*_*s*_ is given according to:

(3) $$\begin{array}{c} \rho_{s}=\frac{1}{\mu_{s}}\left(\frac{\text{TU}}{\alpha }-\mu_{w}\rho_{w}\theta_{r}\right), \end{array}$$

where *α* (cm) is the straight line angular coefficient obtained during CT calibration and *θ*_*r*_ (cm^3^ cm^−3^) represents the residual soil water content.

For each sample, four density matrices were generated from the *μ*_es_ average values for each interval of thickness and collimator. The program Microvis [17] was used to reconstruct and analyze CT images.

#### 2.5.3. Representative Elementary Area

For the REA analysis, *ρ*_*s*_ was calculated for different areas selected from CT images. Firstly, an area comprising almost the whole image was selected, with an irregular shape, named free area (FA). The *ρ*_*s*_ value obtained in the FA of each clod with the theoretical *μ*_*s*_ value was adopted as a reference value. Then, the largest rectangular area possible was delimited inside the sample, without the interference of boundaries in CT images. Within this, consecutive concentric quadrangular areas were selected. The initial area was obtained from a 1 × 1 square matrix (1.1 mm × 1.1 mm). The number of delimited areas within each sample varied according to its size and shape. Further details regarding the procedure carried out can be found in Borges et al. [18].

*ρ*_*s*_ measurements through the traditional sealed clod method for the clayey soil presented CV with 4% variation [19]. Thus, for each sample the REA was determined as a function of *ρ*_*s*_ with the *μ*_es_ average values calculated from the REL results, according to the following criteria:

- relative difference between the *ρ*_*s*_ reference value and each of the remaining areas is not superior to 4%;
- at least three consecutive areas cannot differ from each other regarding *ρ*_*s*_ values, using the variation criterion in item (i).

The optimal thickness value (x*) for the physical properties measurements of the soil samples under study was also calculated and compared to the result obtained via REA analyses [6]. For that, in (4), the average bulk density value was obtained from the *ρ*_*s*_ result for each of the 18 clayey soil clod samples, as well as the *μ*_*s*_ and *μ*_*w*_ theoretical values (XCOM) for the 60 keV energy:

(4) $$\begin{array}{c} x^{*}=\frac{2}{\mu_{s}\rho_{s}+\mu_{w}\rho_{w}\theta_{r}}. \end{array}$$

## 3. Results and Discussion

### 3.1. Soil Mass Attenuation Coefficient

In Table 1 the *μ*_*s*_ values calculated (XCOM) and measured as well as the relative differences amongst them are presented. Errors associated with the measurements in (2) were obtained at the 5th decimal place for the ^137^Cs source and at the 4th decimal place for the ^241^Am source.

Results obtained via XCOM revealed that *μ*_*s*_ values for low energy gamma photons (*≈*60 keV) present bigger difference between the soils under analysis in relation to *μ*_*s*_ values for the radiation energy *≈*662 keV. This is due to the fact that, at low energies, differences in chemical composition affect more significantly the attenuation of a given material [6]. In this case, the clayey soil presented a *μ*_*s*_ value 25.4% higher in relation to the sandy one for the ^241^Am energy. Regarding the ^137^Cs source, results are very close, however, with a slight inversion. This fact is due to the higher amount of Fe_2_O_3_ present in the clayey soil (Table 2) [14]. A simulation carried out with hypothetical values revealed that by increasing the Fe_2_O_3_ amount present in the soil composition, the *μ*_*s*_ for ^241^Am energy increases in some tenths, while for ^137^Cs it is reduced in thousandths.

Regarding the experimental results in Table 1, for the ^137^Cs source it could be observed that for 3 and 4 mm collimators the RDs between *μ*_es_ average values of larger thicknesses in relation to the theoretical *μ*_*s*_ value were below those of smaller thicknesses. A contrary behavior was observed only for the 2 mm collimator, for both soils. Considering the ^241^Am source and 2 and 3 mm collimators, there was no spectrum definition for thicknesses larger than 12 and 14 cm, respectively. The smallest RDs were obtained for *x* < 10 cm and 2 mm collimator (both soils) and 3 mm collimator and sandy soil. For the remaining cases, the lowest RDs were observed at the thicknesses *x* ≥ 10 cm.

The closest *μ*_es_ average values for both thickness intervals and for both soils were obtained with the 2 mm collimator and ^137^Cs source and the 4 mm and ^241^Am source. That is, the differences between thickness intervals are more pronounced when larger collimators are used with the ^137^Cs source and smaller collimators and the ^241^Am source. Regarding the extremely high *μ*_es_ RD for *x* ≥ 10 cm, with ^241^Am source, 2 mm collimator, and clayey soil, the indefinite spectrum for thicknesses over 12 cm is an indication of the unsuitability of these measurements carried out with low energy photons and very small collimators. Soil heterogeneity, its particle size and some compaction of the sample that might occur within the container are possible sources of error [6].

### 3.2. Representative Elementary Length

**Figure 1:**
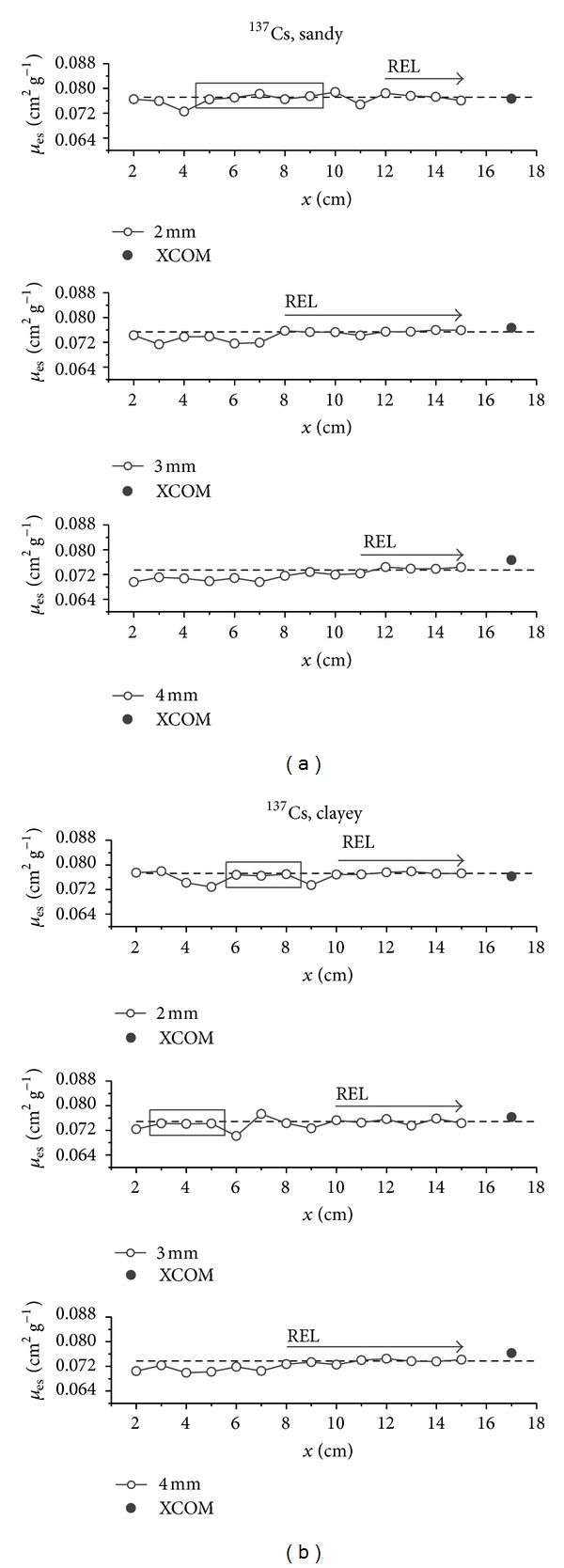
Soil experimental mass attenuation coefficient (*μ*_es_) and representative elementary length (REL). Results were obtained for different sample thicknesses (*x*), types of soil ((a), (b)), and collimators for the ^137^Cs source. The dashed line corresponds to the average of *μ*_es_ values for the sample thickness *x* ≥ 10 cm. The solid symbol corresponds to the theoretical value obtained via XCOM.

**Figure 2:**
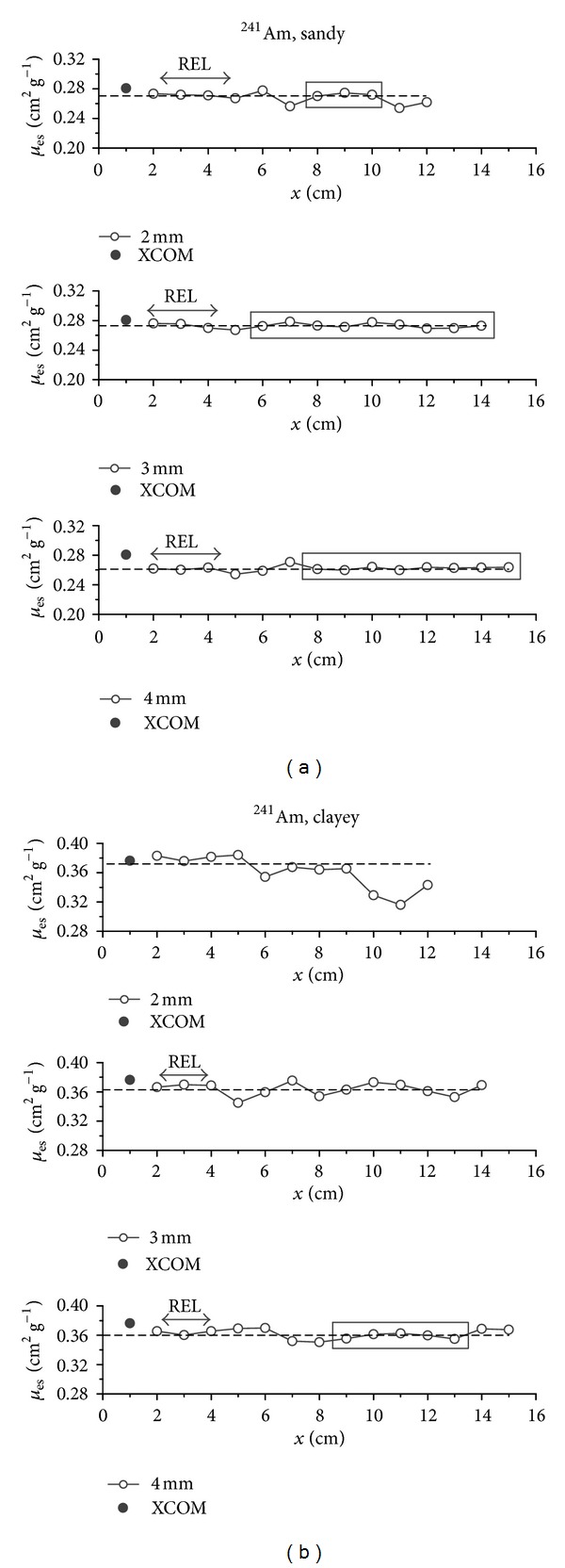
Soil experimental mass attenuation coefficient (*μ*_es_) and representative elementary length (REL). Results were obtained for different sample thicknesses (*x*), types of soil ((a), (b)), and collimators for the ^241^Am source. The dashed line corresponds to the *μ*_es_ average values for the sample thickness *x* < 10 cm. The solid symbol corresponds to the theoretical value obtained via XCOM.

In Figures 1 and 2, the graphs of *μ*_es_ values obtained for each sample thickness under evaluation are presented. From the results variability analysis, it was possible to identify* plateau* regions in the graphs. In general, for the ^137^Cs source, higher fluctuation of each value in relation to the average value is observed (dashed line) for smaller thicknesses, when compared to results obtained for larger thicknesses (Figures 1(a) and 1(b)). Such fluctuations are reduced while the thickness increases up to the point where it reaches the *plateau*, where each *μ*_es_ value presents variation lower than 2% in relation to the average value.

For the sandy soil, the *plateau* occurred from the thicknesses 12, 8, and 11 cm, for collimators 2, 3, and 4 mm, respectively, (Figure 1(a)). For the clayey soil, these values were 10 cm for the 2 and 3 mm collimators and 8 cm for the 4 mm collimator (Figure 1(b)).

For the sandy soil and 2 mm collimator, besides the *plateau*, one more region of stabilization was observed for a minimum number of 3 sequential thicknesses: 5–9 cm. The other collimators, however, did not present the same behavior. For the clayey soil, these regions were observed for the two smaller collimators: 6–8 (2 mm) and 3–5 cm (3 mm). However, despite being stable, these regions are isolated and do not present standard behavior regarding different soils or collimators.

Considering the theoretical *μ*_*s*_ plotted in the sequence of experimental results (solid symbol), in general it could be observed that for both soils, the *plateau* region of smaller collimators presented a more pronounced tendency to the theoretical one, when compared to results obtained for the 4 mm collimator, according to RD values presented in Table 1.

As the ^137^Cs source presents intermediate radiation energy (*≈*662 keV), *μ*_es_ values do not differ much one from another for different texture soils. Thus, it is possible to define a minimum thickness value to be adopted for *μ*_es_ measurements of these soils, with each of the collimators. For the 2 mm collimator, the minimum thickness needed for the *plateau* to be reached for both soils was 12 cm. For 3 and 4 mm collimators, these thicknesses were 10 and 11 cm, respectively. Regarding both soils and any of these three collimators, samples with thicknesses ranging from 12 to 15 cm can be used, once these were the ones that presented a stable result at 2% variation in all cases under study.

For the ^241^Am source (Figures 2(a) and 2(b)), the contrary situation is observed. A *plateau* region for initial thicknesses occurred in all cases (different soils and collimators), except for the 2 mm collimator and clayey soil. This exception can be explained by the association of two factors: the chemical composition of this soil and the diameter of the circular collimator used. As seen in Table 2, the clayey soil presents higher amount of Fe_2_O_3_ (79.27% higher than the sandy one), and for the ^241^Am source the differences in sample chemical composition influenced more significantly its *μ*_*s*_ value. In this case a higher incidence of underestimated *μ*_es_ values (*≈*64%) was observed in relation to the average value, which occurred with thicknesses above 5 cm.

For the sandy soil (Figure 2(a)), another stabilization region besides the *plateau* in the first thicknesses was observed, for all collimators. However, the most stable results occurred with the larger collimators. Especially with the 3 mm collimator, in which case high uniformity between *μ*_es_ values was observed, and only one thickness, 5 cm, presented higher variation. However, this difference was only 0.09 higher than 2%. Regarding the clayey soil, this behavior was only observed with the 4 mm collimator. These stable values observed for larger thicknesses can be explained due to the Compton scattering becoming relevant in the radiation intensity ratio (*I*/*I*_0_) and generating higher results than those expected for low energy photons [6].

In general, the *plateau* regions which were closer to the *μ*_*s*_ value occurred with the smallest collimators. The 4 mm collimator presents higher homogeneity of results and is not so sensitive to variations in the thickness of the sample used and generating more different values in relation to the theoretical ones.

From these results, it could be observed that in the cases in which the *plateau* was reached, the thicknesses 2–4 cm are common to all of them and can be used for representative *μ*_es_ measurements with 2 to 4 mm collimators for the sandy soil and 3 to 4 mm collimators for the clayey one. The possibility to use small samples and, mainly, to obtain representative *μ*_es_ results with them, is important due to the smaller amount needed of a sample to be collected and prepared for use, as well as to the reduction in analysis time.

However, it is important to observe that results presented here were obtained with disturbed samples, which reduces the possibility of occurrence of error due to the nonuniformity of the sample physical structure as, for example, different density regions. The use of undisturbed samples would probably require different thicknesses to obtain representative *μ*_es_ measurements.

### 3.3. Influence of *μ*_*es*_ in REA Measurements for Soil Bulk Density via CT

The soil clod samples presented residual humidity of 0.01 cm^3 ^cm^−3^ and average bulk density of 1.52 ± 0.06 g cm^−3^. The *μ*_*w*_ evaluated was 0.2066 cm^2 ^g^−1^. From these results, the optimal thickness value calculated was 3.48 cm.

With REL results, bulk density matrices were generated with average *μ*_es_ values for *x* ≤ 4 and *x* > 4 cm with 3 and 4 mm collimators. These values were 0.3684 and 0.3623 cm^2 ^g^−1^ (3 mm) and 0.3638 and 0.3611 cm^2 ^g^−1^ (4 mm).

**Figure 3:**
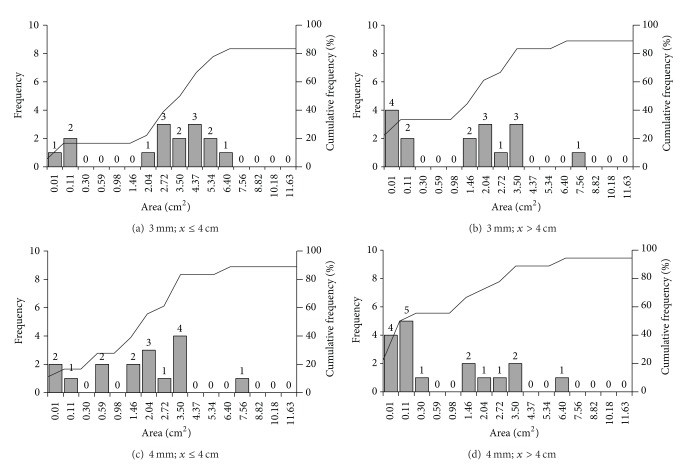
Frequency and cumulative frequency (%) of the number of samples that reached REA for the soil bulk density measurements (*ρ*_*s*_) in each of the areas selected. Results were obtained using the *μ*_es_ average values (*x* ≤ 4 and *x* > 4 cm) for the ^241^Am source and 3 and 4 mm diameter circular collimators.

Figure 3 shows the number of samples that reached REA for each of the cases analyzed. Regarding *μ*_es_ average value obtained for thicknesses  *x* ≤ 4 cm and 3 mm collimator (Figure 3(a)), a total of 15 samples (83%) reached REA up to the 12th quadrangular area selected (6.40 cm^2^). Taking the same collimator and average *μ*_es_ for *x* > 4 cm, 16 samples reached REA up to the 13th area (7.56 cm^2^). With the 4 mm collimator (Figures 3(c) and 3(d)), 16 and 17 samples reached REA up to the 13th and 12th areas, for *x* ≤ 4 and *x* > 4 cm, respectively.

Results revealed that in all cases under analysis over 80% of the samples reached REA within the maximum quadrangular area selected (11.63 cm^2^), which corresponds to a thickness 3.41 cm. This result confirms the x* value calculated, as well as results found in the literature for *ρ*_*s*_ measurement of undisturbed soil samples [6, 20]. However, from the REA analyses it could be observed that most of the samples presented stable *ρ*_*s*_ values for analyses via CT in thicknesses below 3.41 cm. The maximum thicknesses needed for the samples that presented stabilization to reach REA were 2.53 (Figures 3(a) and 3(d)) and 2.75 cm (Figures 3(b) and 3(c)).

According to Miyazaki [3], one of the criteria to define RES is that it should provide a method of measurement which is operationally convenient. The possibility to use samples with thicknesses such as the ones obtained in this study favors the collection and preparation stages and, mainly for analyses via CT, demands lower computational cost.

From Figure 3, an increase in the number of samples which present stable *ρ*_*s*_ values in the first 5 areas can also be seen, when the *μ*_es_ average value is used for *x* > 4 cm. This number was equal to the double of the value for *x* ≤ 4 cm for both collimators: from 3 to 6 (3 mm) and from 5 to 10 samples (4 mm). Thus, results obtained from a nonrepresentative *μ*_es_ value might lead to the use of samples with a smaller size than the ideal one, generating *ρ*_*s*_ values that do not represent the situation found in the experimental field.

**Figure 4:**
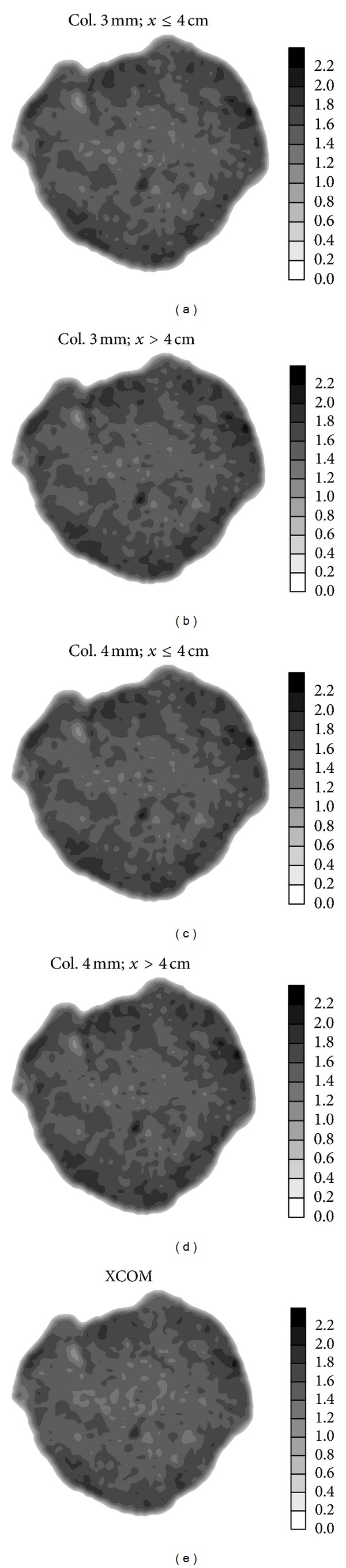
Tomographic images of a clayey soil clod generated with the theoretical value (XCOM) and the *μ*_es_ average values (*x* ≤ 4 and *x* > 4 cm) for the ^241^Am source and different circular collimator (Col.) diameters.

In Figure 4, 2D tomographic images are presented which were generated from one of the clayey soil clods data used in this study and with different *μ*_es_ average values. Qualitative analyses indicate *ρ*_*s*_ variations inside the sample. According to the *μ*_es_ value used, higher or lower density regions (darker or lighter shades in the grey scale) might increase or be reduced in the image, leading to *ρ*_*s*_ over- or underestimation.

Figures 4(a) and 4(c) correspond to images that present *ρ*_*s*_ distributions closer to those obtained with the *μ*_*s*_ theoretical value. This result is confirmed by the deviation between these *μ*_es_ average values (*x* ≤ 4 cm) and the theoretical value. The RDs were 2.13 and 3.75% (3 mm) and 3.35 and 4.06% (4 mm) for thicknesses *x* ≤ 4 and *x* > 4 cm, respectively. That is, the lowest deviations were obtained for the average of the smallest thicknesses with both collimators.

## 4. Conclusions

Results confirmed that for best accuracy in *μ*_es_ measurements the sample thickness might be optimized. For measurements of sandy and clayey soils, with circular collimators of 2, 3, and 4 mm diameter, the REL lies in the thickness intervals of 12–15 and 2–4 cm for the sources ^137^Cs and ^241^Am, respectively. These thickness intervals provide *μ*_es_ with the smallest variations and closer to the theoretical ones. The application of the results obtained from source ^241^Am for clayey soil in REA evaluations for *ρ*_*s*_ measurements via CT indicates that *μ*_es_ average values for *x* > 4 cm might induce to the use of samples with an unrepresentative size.

## Figures and Tables

**Table 1:** Soil mass attenuation coefficients (*µ*_*s*_) calculated (XCOM) and measured.

Collimator		^ 137^Cs				^ 241^Am			
		Sandy		Clayey		Sandy		Clayey	
		*µ*_*s*_ (cm^2^ g^−1^)	RD (%)	*µ*_*s*_ (cm^2^ g^−1^)	RD (%)	*µ*_*s*_ (cm^2^ g^−1^)	RD (%)	*µ*_*s*_ (cm^2^ g^−1^)	RD (%)
	XCOM	0.0767	—	0.0763	—	0.2807	—	0.3764	—
2 mm	<10*	0.0763	0.5	0.0758	0.7	0.2703	3.7	0.3720	1.2
	≥10	0.0772	0.7	0.0773	1.3	0.2626	6.4	0.3295	12.5
3 mm	<10	0.0735	4.2	0.0737	3.4	0.2730	2.7	0.3628	3.6
	≥10	0.0754	1.7	0.0748	2.0	0.2728	2.8	0.3652	3.0
4 mm	<10	0.0708	7.7	0.0714	6.4	0.2612	6.9	0.3611	4.1
	≥10	0.0735	4.2	0.0737	3.4	0.2629	6.3	0.3625	3.7

*Experimental results correspond to the average values for the thicknesses *x* < 10 and *x* ≥ 10 cm for each energy, collimator, and soil. The relative differences (RDs) were calculated between each average value and the respective *µ*_*s*_ obtained via XCOM, adopted as a reference value.

**Table 2:** Elements present in amounts over 1% in the soils under analysis.

Soil	Chemical components (%)					
	SiO_2_	Al_2_O_3_	Fe_2_O_3_	TiO_2_	SO_3_	Others
Sandy	62.80	30.03	3.47	1.93	1.39	0.38
Clayey	44.19	32.84	16.74	3.49	1.71	1.03
